# The Effect of the Operation of Downhole Equipment on the Processes of Corrosive Wear (by the Example Inflow Control Devices of Nozzle Type)

**DOI:** 10.3390/ma15196731

**Published:** 2022-09-28

**Authors:** Pavel Kruk, Ivan Golubev, Nikita Shaposhnikov, Julia Shinder, Dmitry Kotov

**Affiliations:** 1 Scientific and Technological Complex “New Technologies and Materials”, Institute of Advanced Engineering Technologies, Peter the Great Saint-Petersburg Polytechnic University, Polytechnicheskaya 29, 194064 St. Petersburg, Russia; 2 LLC “SAND CONTROL LAB”, Revolution Highway 88 Letter K, Office 2H, 195279 St. Petersburg, Russia

**Keywords:** CO_2_ corrosion, erosion, rotating cage autoclave (RCA), nozzle, inflow control device (ICD), wall shear stress, well completion design

## Abstract

Existing approaches to assessing the reliability and safe operation of downhole equipment provide for assessing only the direct impact of erosion and corrosion processes on the degradation of the material. At the same time, the influence of downhole equipment operating modes on the intensity of corrosion and erosion processes is not evaluated. The necessity of using inflow control devices is shown. The necessity of selecting inflow control devices for specific operating modes of the well is highlighted in order to avoid increased erosion wear due to increased sand production. In this article, a series of studies was conducted to assess the influence of the hydrodynamic characteristics of the fluid flow, which vary depending on the operating modes of the nozzle-type inflow control device, on corrosion processes in the well. It was shown that the level of wall shear stress (WSS) of the base pipe, immediately after the flow control device in the direction of fluid flow, affects the intensification of corrosion processes in downhole equipment. Studies of WSS on the pipe wall were carried out using a dynamic autoclave and elements of mathematical modeling. The design factors (the angle of inclination of the main hydraulic channel of the inflow control device relative to the base pipe) affecting the change in tangential stresses on the wall of the base pipe were investigated. The mechanism of corrosion wear of the base pipe was revealed.

## 1. Introduction

The selection of the optimal set of downhole equipment is one of the most important stages in the design and construction of wells, given the important fact that most of the equipment for completing oil and gas wells is not recoverable. This is especially true in the presence of such complicating factors as significant sand production in terrigenous poorly consolidated reservoirs, strong geological stratification of the deposit, the presence of closely spaced water and gas–oil contact, a strong heterogeneity of reservoir properties, and the presence of conditions for intensive corrosion processes. In the studies [1,2,3] carried out on the topic under consideration, in most cases, only the one-sided influence of corrosion processes on the operation of downhole equipment was described, and the effect of the operation of downhole equipment on corrosion processes inside the well was not considered. However, the effect of downhole equipment operation on downhole corrosion can be catastrophic. Examples of this include the leaking completion pipe in the Giant Offshore field located 84 Km NW of Abu Dhabi Island. Corrosive environments and the installation of inflow control devices (ICDs) of the nozzle type have resulted in a wear-through of the completion pipe in this field [4]. While studying this phenomenon, the authors gave the main recommendation to replace the grade of pipe material with a more corrosion-resistant one. In [5], the authors focused on the corrosion resistance of the ICD itself and did not pay proper attention to the influence of the ICD on the corrosion wear of the base pipe and methods of combating it, except for the change in the tube material. In this regard, the authors believe that it is necessary to more fully investigate the mechanisms of corrosion wear of the base pipe under the influence of ICD operation, as well as to develop more effective and less costly methods to reduce the level of corrosion wear of the base pipe than replacing the material of which it is made.

## 2. Materials and Methods

### 2.1. Formulation of the Problem

#### 2.1.1. Determining the Impact of Downhole Equipment on Erosion Corrosion Processes

Having analyzed the parameters that affect the course of corrosion processes (the proportion of water in the flow, the presence and concentration of CO_2_ and H_2_S, the type of flow, and the tangential stresses it creates on the wall) [1] and erosion processes (the volume of mechanical impurities in the flow, the particle sizes of mechanical impurities, speed flow, etc.) [2], the parameters that have the greatest impact on the operation of downhole equipment were identified.

The list of typical downhole completion equipment includes a liner hanger and packer, annular packers of various types, downhole sand screens with retrofit in the form of inflow control devices, centralizers, shoe and/or couplings with a check valve and casing.

From the analysis of the impact of the operation of the main downhole equipment on the intensity of corrosion and erosion processes (Table 1), the device that affects the largest number of factors is the inflow control device. It is also worth noting that various downhole equipment has a different degree of influence and the possibility of correcting this influence on various given factors. Thus, only the inflow control device can have a significant impact on the proportion of water in the flow and the volume of mechanical impurities. At the same time, inflow control devices can have a significant impact on changing the flow rates and regimes, which in turn has a great impact on the intensity of corrosion and erosion processes. Therefore, in this article, the influence of the inflow control devices on the erosion–corrosion processes was considered.

#### 2.1.2. Inflow Control Devices

An inflow control device (ICD) is a piece of well completion equipment that is used to evenly distribute inflow along the wellbore. There are several different types of ICDs (channel type, nozzle type, autonomous type, etc.). Despite the variety of designs, the principle of operation of various inflow control devices is the same—flow restriction by creating an additional pressure drop between the reservoir and the well to achieve a uniform distribution of the inflow profile. In this study, the authors considered, as an example, an ICD of the choke type, as the simplest and most common in practice. With a more even distribution of the flow profile, water or gas coning, sand production, and other drawdown problems can be solved. Inflow control devices can play both positive and negative roles in well performance [6]. Understanding the positive and negative factors of the influence of the ICD on various well operation parameters, including corrosion and erosion processes, can help to correctly minimize the negative impact of the ICD operation on downhole processes and enhance the positive aspects of this influence.

Below are considered all the factors affecting erosion and corrosion processes individually and in combination, in relation to the effect of the modes of operation of inflow control devices on them.

#### 2.1.3. The Proportion of Water in the Flow and the Removal of Mechanical Particles

Field data show that the amount of sand in terrigenous reservoirs tends to increase when the water content in the produced fluids increases [7,8].

**Figure 1:**
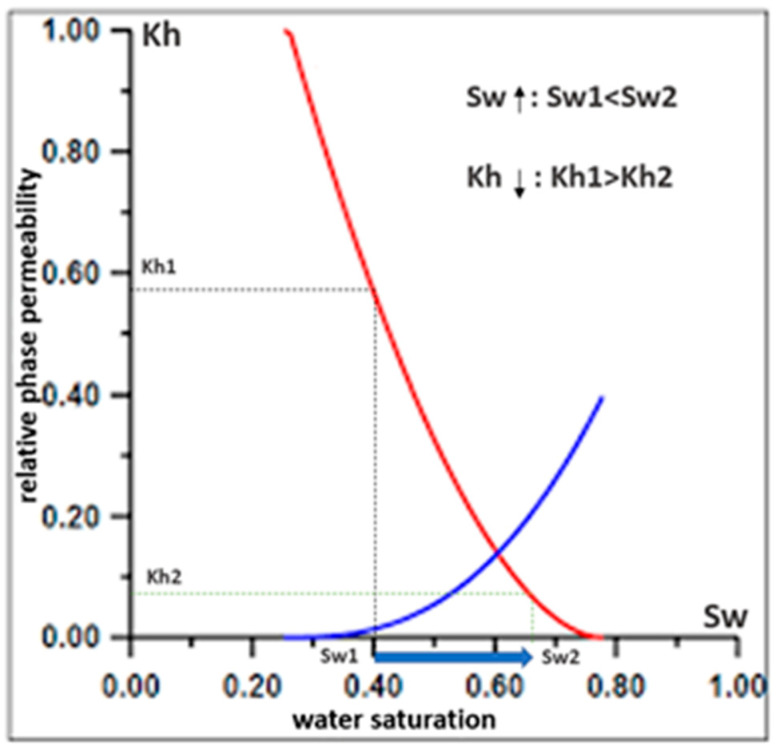
The effect of well water cut increase on sand production increase. Kh, relative phase permeability; Sw, water saturation.

There are two main reasons for the increasing water content in an oil and/or gas well—water inflow from adjacent injection wells and water breakthrough from the aquifer zone (underlying water zone). An increase in the water content in the near-wellbore space affects the adhesion of rock particles to each other. This bonding is provided by the surface tension of the bound water, and it decreases as the particles adhere to the formation water. This reduces the cohesive ability of the rock particles. Moreover, as the water cut increases, the relative permeability for oil decreases. To maintain the design oil flow rate under these conditions, it is necessary to increase the drawdown at the bottom of the well (Figure 1) (Joshi formula Equations (1) and (2) [9]). 

The blue line in Figure 1 shows the dependence of relative phase permeability on saturation with water, the red line shows the dependence of relative phase permeability on oil saturation.

(1) $$q_{гc}=\frac{2\pi k_{h}h\left(P_{k}-P_{c}\right)}{\mu B_{0}(\ln\frac{a+\sqrt{a^{2}-{\left(L/2\right)}^{2}}}{L/2}+\frac{h}{L}\ln\frac{h}{2r_{w}})}$$

where $q_{гc}$—oil flow rate of a horizontal wellbore; *k_h_*—horizontal permeability; *h*—oil-saturated thickness; *P_k_* − *P_c_*—reservoir pressure drop; *µ*—oil viscosity; $B_{0}$—oil volume factor; *L*—length of the horizontal section of the well; *r_w_*—wellbore radius;

(2) $$a=0.5{\left(0.5+\sqrt{0.25+{\left(1/\left(L/2r_{w}\right)\right)}^{4}}\right)}^{0.5}$$

An increase in drawdown at the bottom of the well leads in turn to an increase in the shear force of sand particles relative to each other. A decrease in cohesiveness and an increase in shear force increase the likelihood of the removal of the mechanical particles of the rock. [10] The removal of mechanical particles has a direct impact on the rate of the erosion process in the pipe walls [11]. All the main modern models of erosive wear rate—DNV [12] and Tulsa E/CRC [13]—use sand consumption as one of the main parameters affecting the erosion process. Thus, an increase in water cut and sand production has a strong impact on erosion processes in the well.

One of the main functions of the ICD is to reduce the risks of water cut and thereby reduce the risk of sand production, namely, to reduce the intensity of downhole erosion processes in general.

#### 2.1.4. Shear Stresses on the Wall

With a positive effect of inflow control devices on reducing sand production and reducing water cut (and, as a result, on reducing the intensity of corrosion and erosion processes inside the well), there is also a negative side of the effect of ICDs on corrosion processes due to an increase in shear stresses on the pipe wall.

Alvarez [14] revealed and proved the influence of the nozzle-type ICD on the corrosive wear of the pipe wall after (relative to the flow direction) the ICD was placed in it. The authors gave only general recommendations for minimizing or eliminating the effect of nozzle-type ICDs on the development of corrosion processes.

**Figure 2:**
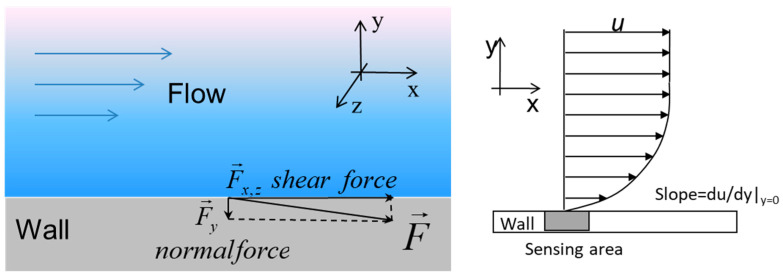
The mechanism of formation of shear stresses from the interaction of the flow with the wall.

When analyzing the factors that influenced the wear of the pipe wall, it is possible to distinguish the factors of the content of various chemicals (water, CO_2_), mechanical impurities (rock particles) in the flow, and the factors of flow dynamics (flow rate, flow nature). It is necessary to determine which group of factors has the greatest influence on the process of corrosion wear. Alvarez [14] showed that failure occurred only in certain sections of the pipe. At the same time, the remaining sections of the pipe, through which the flow passed with the same content of chemicals and mechanical impurities as through the inflow control devices, were not significantly affected by corrosion processes and subsequent destruction. It follows that the flow dynamics factors generated by the inflow control device had the greatest effect on pipe wear. To describe the influence of flow dynamics factors on corrosion processes, the value of wall shear stress (WSS) is usually used as the most comprehensive indicator of such an influence [15,16]. WSS (τ, N/m^2^) is the force per unit area of the pipe wall due to fluid friction. Tangential stress at the wall is the result of the influence of the velocity gradient in the near-wall region and the dynamic viscosity of the fluid (Figure 2).

Based on the previous points, we conducted this study to identify the effects of fluid flow hydrodynamic characteristics on corrosion processes in the well. WSS is the basic value for estimating the influence of fluid flow characteristics on corrosion processes.

### 2.2. Research Methodology

To identify the mechanisms of the effect of the operation of the nozzle-type inflow control device (ICD) on the corrosion process and to develop specific approaches to minimize this effect, it is necessary to determine the rate of the corrosive wear of the pipe wall section immediately after (relative to the flow direction) the nozzle-type ICD.

To study the corrosion rate in laboratory conditions, various test methods are used [17,18].

When choosing a test method, the authors compared various test methods to determine the corrosion rate in laboratory conditions [19]. Based on the results of a quantitative comparison of the general and pitting corrosion rates obtained in the laboratory with the indicators obtained in similar pipeline conditions, S. Papavinasam et al. [18] ranked the corrosion rate test methods in the laboratory.

Based on the results of research by S. Papavinasam et al. [19], the corrosion rate determination was carried out according to the method with the highest-rank rotating cage autoclave (RCA) test in an autoclave with a rotating agitator [20].

The parameters of wall shear stress (WSS) can be obtained either by an analytical method with the averaging of parameters over velocities and flow rates or by using computer hydrodynamic modeling methods. Since the authors of the article considered a local corrosion model and the geometric dimensions of the objects under consideration were complex and the scatter in size was quite large (from the nozzle hole of 1.4 mm to the considered section of the pipe space under the ICD of 1–2 m), there was a fear that the analytical methods of calculations with rough averaging can distort the picture of ongoing processes. Therefore, to reproduce the parameters of WSS in the autoclave experiment, the authors of this article carried out the computational fluid dynamics (CFD) of a pipe section with an installed ICD in the Ansys Fluent software package.

To carry out the hydrodynamic calculations of a pipe section with an ICD, all the necessary input parameters (flow rates, pressures, etc.) were obtained from the model of operation of a nozzle-type ICD in a well. This model was compiled based on the results of the hydrodynamic tests of the ICD on the flow loop test bench and from the initial data from the reference field.

Thus, the general methodology for conducting a study consists of several successive steps:

1. Formation of initial data on operating modes and fluids from a real field;
2. Conducting hydrodynamic tests of the ICD to determine the characteristics;
3. Calculation of operating modes of the ICD in the well by the method of nodal analysis (Appendix A);
4. Computer simulation of CFD in a pipe section with an installed ICD;
5. Conducting laboratory experiments to determine the corrosion rate in an autoclave with a rotating stirrer;
6. Examination of samples after the gravimetric test, examination of samples with a scanning electron microscope, determination of corrosion rate, determination of the nature of surface damage, and formation of corrosion products;
7. Analysis of the results and drawing conclusions about the influence of wall shear stress levels on the intensification of corrosion processes in downhole equipment.

### 2.3. Description of the Stage of Numerical Simulation of the Hydrodynamics of the Nozzle Operation

Numerical simulation of the turbulent flow of a viscous incompressible fluid was carried out using the ANSYS Fluent software package. A two-dimensional problem in a vertical section of a pipe was considered. The pipe diameter was chosen to be 0.1 m, and the nozzle’s hole diameter was 1.4 mm. The computational domain included a pipe section 2 m long to eliminate the influence of boundary conditions on the nature of the fluid flow in the nozzle location area. A structured computational grid was used with thickening toward the pipe walls for an accurate calculation of WSS. Turbulence modeling was carried out using Menter’s shear stress transport (SST) model. The fluid density was set equal to 998 kg/m^3^, and viscosity was set to 6 cP.

Mathematical modeling was carried out for a pipe without a nozzle, as well as for pipes with three angles of the nozzle hole: 90. The fluid velocity at the nozzle inlet in all variants was 0.53233 m/s. Two different values of the fluid velocity at the pipe inlet were considered: 0.662 m/s and 0.297 m/s.

### 2.4. Setting up the Autoclave Experiment

#### 2.4.1. Experimental Devices

**Figure 3:**
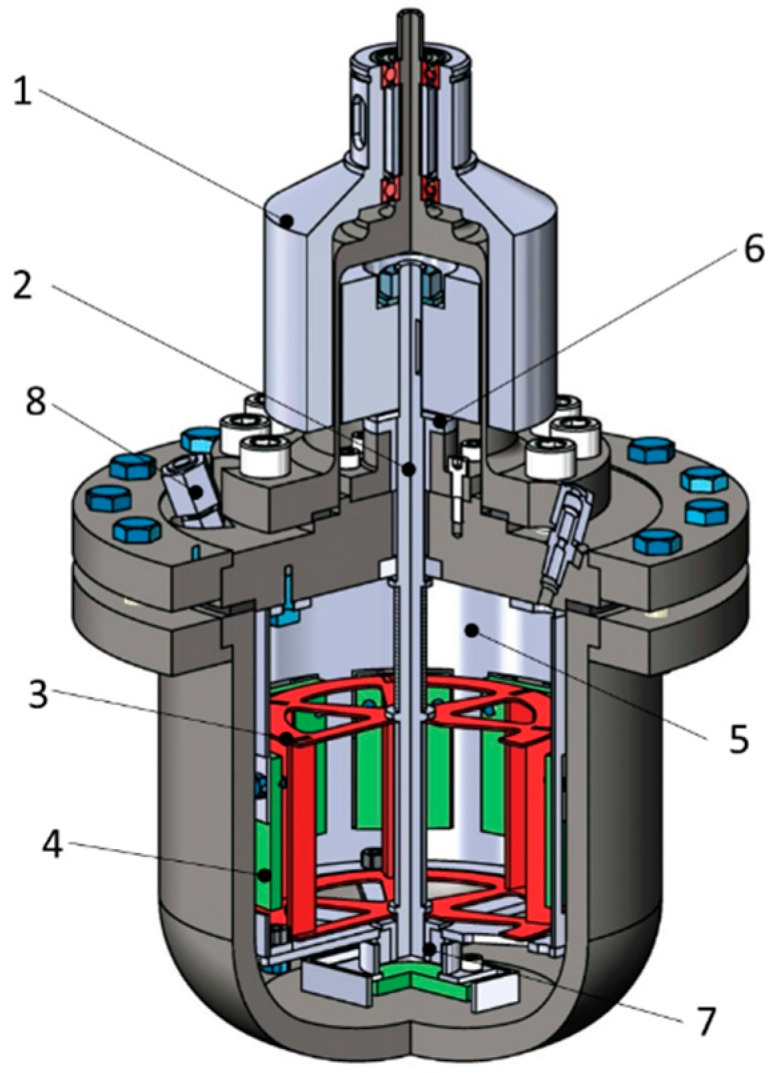
Design of the modified RCA method autoclave. 1—Magnetic Coupling; 2—Shaft; 3—Cage; 4—Samples; 5—Drum; 6, 7—Plain Bearings; 8—Fittings.

This paper proposes testing for the carbon dioxide (CO_2_) corrosion of the material of the liner pipe on the inner wall which is affected by the flow from the nozzle-type ICD installed in the wall of the pipe, a method of laboratory autoclave tests with a stirrer. The technique is based on the rotating cage autoclave (RCA) test method [20] with several modifications. The design of the autoclave is shown in Figure 3. The torque from the electric motor is transmitted through a magnetic coupling (1) directly into the autoclave to the shaft (2). The movement of the flow in the autoclave is created by imparting a rotational movement of the liquid by the cage (3). Nine samples (4) are fixed in the drum (5); they are on the same level as the cage. To reduce losses and increase the service life, the shaft is fixed in plain bearings (6.7). Three fittings (8) are provided for the inlet and outlet of gases.

Temperature and pressure measurements in the autoclave were carried out using the DTPK011-0.512, OVEN (Ivanovo, Russia) temperature sensor and the TM-320P, ROSMA (Nizhny Novgorod, Russia) pressure gauge.

#### 2.4.2. Experimental Materials

The test environment consisted of two phases:

Gas phase. As a model, CO_2_ gas with its partial pressure in the well was taken. The average mole fraction of CO_2_ in the fluid (nCO_2_) was 0.425 mol%. The partial pressure of CO_2_ in the well was equal to Pst × nCO_2_. The average reservoir pressure and the corresponding CO_2_ partial pressure across the reservoirs are shown in Table 2.

Water phase (reservoir water). Mineralized water with a mineralization of 4.68 g/L was taken as a model of the aqueous phase. The composition of the model environment is shown in Table 3.

#### 2.4.3. Experimental Processes

Before the start of the tests, samples of the test material were prepared and placed in the appropriate places in the autoclave drum. The witness samples were plates measuring 5 × 3 cm and 0.5 cm thick with 2 holes for mounting in an autoclave. The surface area **of** the samples was 38 cm^2^. The plates were made of steel sheets, a steel analog of materials for borehole pipes/casing columns.

The autoclave was filled to 90% with mineralized water; the remaining 10% was the gas phase in a ratio that provides a partial pressure of carbon dioxide equal to its pressure at the wellhead.

The test temperature was 64 °C.

The total pressure in the autoclave was 6 MPa.

Testing time—48 h.

pH—7.90.

The pH level was measured using pH-410 instruments with a measurement error of ±0.05.

After that, the agitator was switched on for the specified test time. The agitator rotation speed was determined based on the results of the mathematical modeling of flows in the well segment with an installed nozzle-type inflow control device. Mathematical modeling was carried out at a flow velocity of 0.662 m/s at the pipe inlet and 0.53233 m/s at the nozzle inlet. WSS on the pipe wall immediately after the inflow control device in the direction of the fluid flow is shown in Table 4.

Before and after autoclave tests, samples were weighed on laboratory electronic scales CE224-C, SARTOGOSM (Russia) with an accuracy of 0.001 g.

### 2.5. Setting up the Scanning Electron Microscopy (SEM) Sample Testing

The most representative samples from each group were selected from the test samples: 50, 500, and 720 rpm with sample numbers 4, 2, and 7, respectively. Then, sections were cut from each sample to prepare thin sections. Thin sections were examined with a Supra 40 VP scanning electron microscope (SEM), Karl Zeiss (Jena, Germany). The microscope is equipped with the system of computer control of electron beam scanning and digital registration of signals and images, X-ray spectral microanalyzer INCA WAVE, and INCA X-MAX.

## 3. Results

### 3.1. Results of Numerical Simulation of the Hydrodynamics of Choke Operation

**Figure 4:**
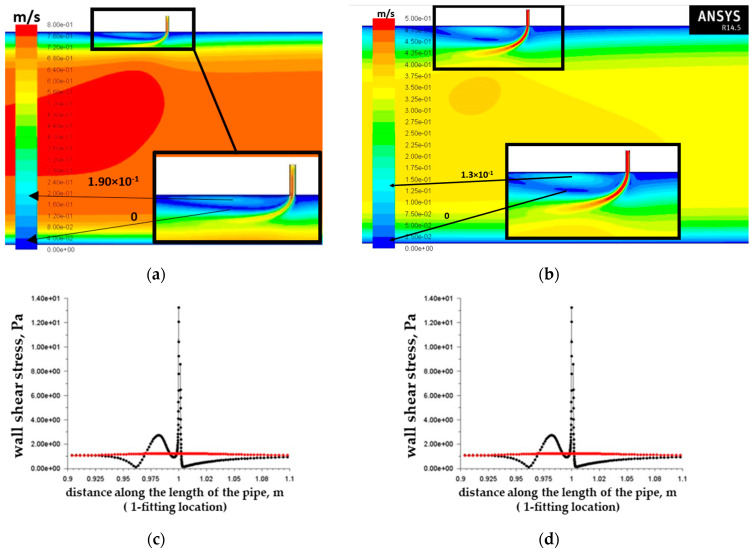
Velocity modulus field in the vertical section of the pipe for the variant of the nozzle-type ICD channel inclination angle of 90° and fluid velocity at the pipe inlet of 0.662 m/s (**a**) and 0.297 m/s (**b**); graphs of shear stresses on the pipe walls at 0.1 m from the nozzle of fluid velocity at the pipe inlet of 0.662 m/s (**c**) and 0.297 m/s (**d**). The black lines correspond to the wall on which the nozzle-type ICD is located, and the red lines correspond to the opposite wall.

Figure 4a,b show the zones of a local increase in flow rates near the wall of the base pipe immediately behind the inflow control device. Such a local increase in the flow velocity in the wall zone, which appeared as a result of the hydrodynamic influence of the flow passing through the inflow control device on the main flow in the pipe, had a direct effect on the local increases in tangential stresses on the wall of the base pipe indicated in the graphs of Figure 4c,d.

For further tests, the variant with the highest tangential stresses on the wall was selected (tangential stresses on the wall 13 Pa, Figure 4c) to study the most dangerous variant of downhole conditions. After autoclave tests (described in Section 3) and evaluation of their results (sufficiently high rates of erosive wear of 1.025 mm/year, Table 5), the authors of the article decided to conduct additional studies of the influence of the design of the inflow control device to reduce the level of tangential stresses on the wall and the level of corrosion wear of the base pipe. 

The results obtained in Table 5 show an increase in the corrosion rate with an increasing speed of the autoclave stirrer and an increase in wall shear stress (WSS).

**Figure 5:**
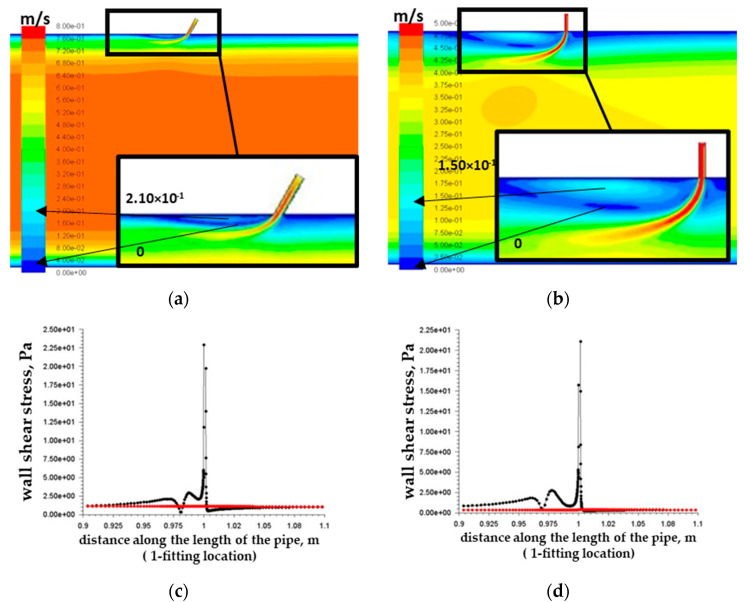
Velocity modulus field in the vertical section of the pipe for the variant of the nozzle-type ICD channel inclination angle of 60° and fluid velocity at the pipe inlet of 0.662 m/s (**a**) and 0.297 m/s (**b**); graphs of shear stresses on the pipe walls at 0.1 m from the nozzle of fluid velocity at the pipe inlet of 0.662 m/s (**c**) and 0.297 m/s (**d**). The black lines correspond to the wall on which the nozzle-type ICD is located, and the red lines correspond to the opposite wall.

**Figure 6:**
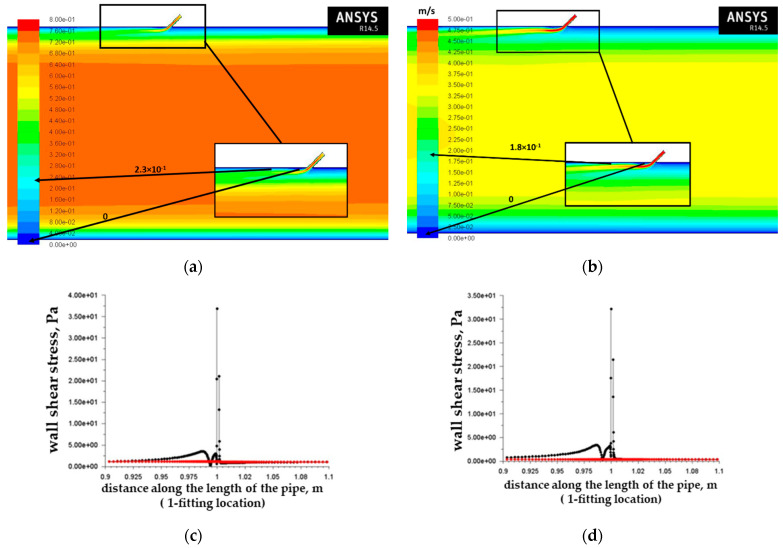
Velocity modulus field in the vertical section of the pipe for the variant of the nozzle-type ICD channel inclination angle of 4 5° and fluid velocity at the pipe inlet of 0.662 m/s (**a**) and 0.297 m/s (**b**); graphs of shear stresses on the pipe walls at 0.1 m from the nozzle of fluid velocity at the pipe inlet of 0.662 m/s (**c**) and 0.297 m/s (**d**). The black lines correspond to the wall on which the nozzle-type ICD is located, and the red lines correspond to the opposite wall.

Having analyzed a number of articles concerning flow modeling in inflow control devices, evaluation of tangential stresses on walls, and optimization of the design of inflow control devices [21,22], the authors came to the conclusion that it is necessary to check the influence of the channel angle of the inflow control device to the axis of the base pipe. This parameter of the design of the inflow control device has the least effect on the operating mode of the inflow control device, but it can greatly affect the nature of the distribution of the flow rate near the wall of the base pipe and the tangential stresses on the wall in the zone after the inflow control device. For further investigation, the angles of inclination of the channel of the inflow control device to the axis of the base pipe in the direction of the arrival of the main flow of 60 and 45 degrees were selected. For these designs, CFD calculations were carried out with completely similar conditions as for the design of the inflow control device with an inclination angle of 90 degrees (Figure 5 and Figure 6).

Having obtained the results of modeling the operation of the nozzle-type ICD with the channel inclination angles of 60 and 45 degrees to the axis of the base pipe for the strongest autoclave tests, the greatest results of tangential stresses on the wall (60 degrees: 23 Pa, Figure 5c; 45 degrees: 36 Pa, Figure 6c) calculations with a flow velocity of 0.662 m/s were shown for each of the tilt angle options. For autoclave tests, the most critical indicator for tangential stresses on the wall was selected: 36 Pa with an angle of inclination of the channel of the nozzle-type ICD to the base pipe of 45 degrees.

### 3.2. Gravimetric Test Results

After the autoclave experiments, the samples were subjected to gravimetric tests to determine the corrosion rates (CRs). Corrosion rates were obtained according to ASTM G31-7222 [23]. In autoclave experiments, three samples were examined for each autoclave rotation speed. After gravimetric analysis, the average corrosion rate (CR) in mm/year of the three samples was calculated for each autoclave rotation speed. Table 4 shows the corrosion rate (CR) mm yr^−1^ calculated using Equation (3):

(3) $$\mathrm{CR}=87.6\frac{W}{\rho *A*t}$$

where W is the weight loss of the substrates in mg, *ρ* is the metal density in g cm^−3^, *A* is the surface area of the substrates in cm^2^, and *t* is the time in hours.

The density of samples is 7.85 g/cm^3^. The testing time and surface area of samples are indicated in Section 2.4.3.

### 3.3. Results of Sample Examination with a Scanning Electron Microscope (SEM)

The scanning electron microscope (CEM) study made it possible to determine the morphology of the corroded surface and qualitative analysis of corrosion products.

#### 3.3.1. Rotational Speed: 50 rpm

**Figure 7:**
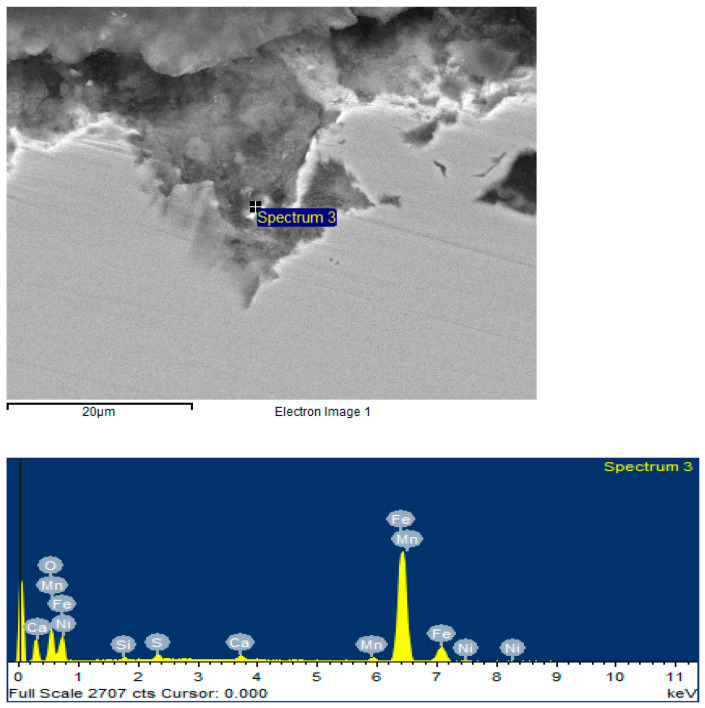
Surface image and elemental composition for the sample (test speed 50 rpm) obtained with CEM.

Traces of general corrosion were found on the surface of the sample. There are areas of pitting corrosion. Qualitative analysis showed the presence of compounds based on oxygen, carbon, and calcium (Figure 7). The products may consist of iron oxides FeO, Fe_2_O_3_, and Fe_3_O_4_; iron carbonates: FeCO_3_; and calcium carbonates: CaCO_3_. Moreover, traces of sulfur up to 1% were found, indicating that one of the corrosion products may be iron sulfide FeS.

#### 3.3.2. Rotational Speed: 500 rpm

**Figure 8:**
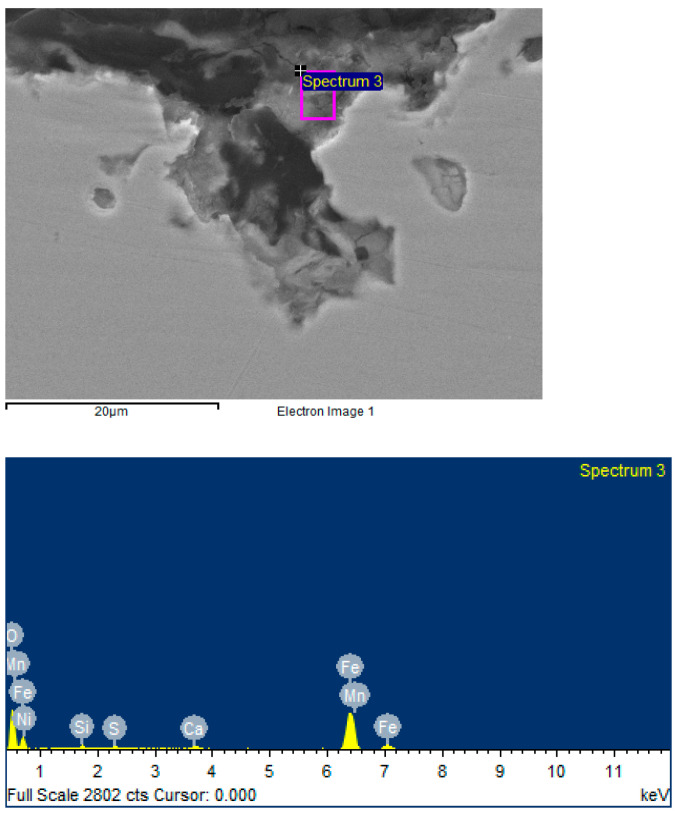
Surface image and elemental composition for the sample (test speed 500 rpm) obtained with CEM.

**Figure 9:**
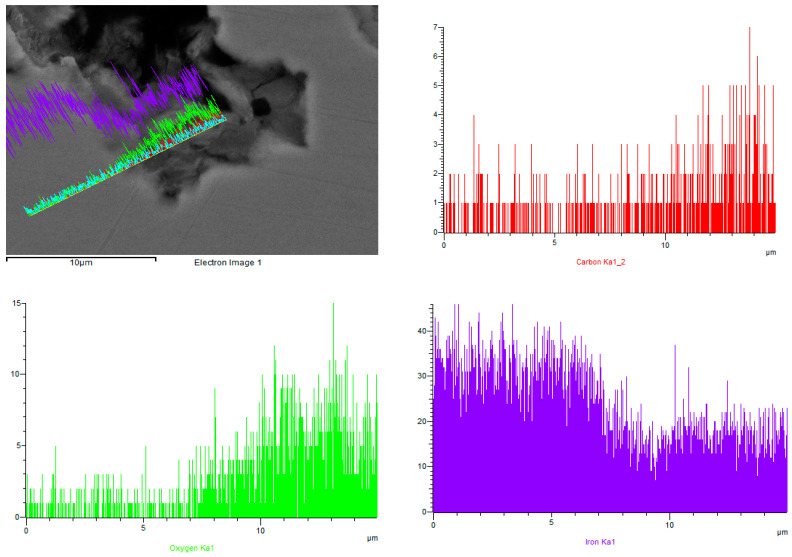
Image of the sample (test speed 500 rpm) surface (top left) and the change in elemental composition as a function of removal for carbon (top right), oxygen (bottom left), and iron (bottom right).

Point scanning of the sample surface allowed us to determine insignificant traces of iron sulfides FeS (Figure 8). Traces of iron oxides and carbonates were detected on the surface. Linear scanning of the area was performed for this sample (Figure 9). This allowed us to understand how the composition changes as a function of distance from the matrix (steel) to the surface of the sample. The intensity of the peaks for carbon and oxygen increased with distance, while the concentration of iron decreased.

#### 3.3.3. Rotational Speed: 720 rpm

**Figure 10:**
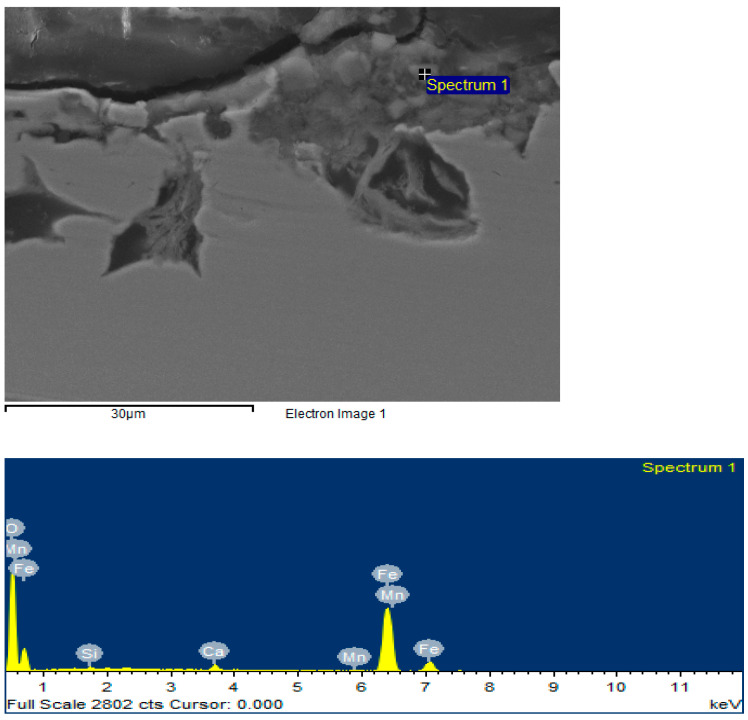
Surface image and elemental composition for the sample (test speed 720 rpm) obtained with CEM.

The corrosion products at this rotational speed also have traces of iron carbonates and oxides. However, traces of sulfides (Figure 10) could not be detected.

## 4. Discussion

### 4.1. Evaluation of the Results of the Experiment

According to the results of measurements on scanning electron microscope (CEM) samples, we can conclude that each of the samples is characterized by general and local corrosion. At 50 rpm, there are significant manifestations of local corrosion, which are due to the presence of stagnant zones with an increased concentration of a corrosive medium on the surface of the sample. At 500 and 720 rpm, general corrosion prevails over local corrosion.

At rotational speeds of 50 and 500 rpm, traces of sulfides are observed (Figure 7 and Figure 8), but at 720 rpm, they are absent (Figure 10). This feature can be explained by the influence of the flow velocity and the abrasive effect of the corrosive medium. At low rotational speeds, a film of corrosion products is formed on the surface of the samples, which is stable at speeds of 50 and 500 rpm. Increasing the rotational speed to 720 rpm results in an interrelated process of corrosion product separation and new product formation, with a significantly lower concentration of the corrosion medium on the surface than at 50 and 500 rpm. However, the time for the formation of this product is limited, and iron sulfides do not have time to form on the surface of the test specimen.

### 4.2. Influence of Wall Shear Stress Levels on the Intensification of Corrosion Processes in Downhole Equipment

**Figure 11:**
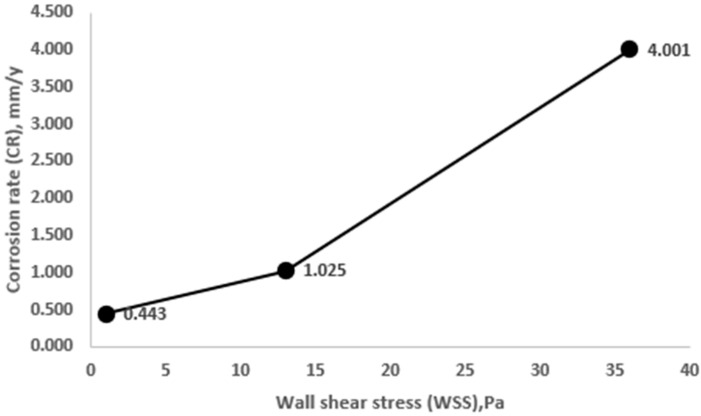
Influence of wall shear stress levels on the intensification of corrosion rate.

Figure 11 shows that the quantitative dependence of the corrosion rate on the tangential stresses on the wall increases almost linearly. A slight increase in the intensity of corrosion at tangential stresses in 36 Pa can be explained by the mechanism of removal of the sulfide film from the surface of the samples with an increase in the flow rate and wall shear stress (WSS), which is confirmed by the data of the CEM analysis of the samples. By the smoothness of the curve and the absence of peaks in the figure, we can indirectly judge the combined influence of corrosion and erosion factors in the process of mass loss and the absence of the effects of the predominance of corrosion or erosion factors, as described by R. S. Prasannakumar [24]. The effect of erosion on the processes of mass loss of the sample was not considered separately in this work. Consideration of erosive wear by the fluid flow together with corrosion processes, as well as erosive wear by mechanical particles, is of great importance for the operation of downhole equipment. The authors intend to carry out work in the near future to account for erosive factors in the processes of corrosion wear.

To determine the practical significance of the identified dependence of the influence of tangential stresses and flow rate on the corrosion rate, it is necessary to connect the parameters of corrosion rate with the parameters of operation of downhole equipment. This will allow an understanding of how the change in flow rate and WSS through the change in corrosion rate can affect the serviceability of downhole equipment.

In order to evaluate the performance and failure-free life of downhole equipment (liner with an installed nozzle-type inflow control device (ICD) in the “N” field), we took data on corrosion rate at different tangential stresses created by the ICD from Table 5. To determine trouble-free lifetime, we considered a case of the penetrating corrosion of the pipe wall with an installed nozzle-type ICD (with a wall thickness of 7.4 mm), at which its tightness will be lost.

No-failure service life of the liner with ICD (y) = Thickness of the pipe wall (mm)/Av. corrosion rate (mm/y) (from Table 6).

The results of the calculation of the no-failure service life of the liner with the installed nozzle-type ICD in the N field are presented in Table 6.

Table 6 shows that with a decrease in the angle of inclination of the channel of the inflow control device from 90 degrees to 40 degrees, the corrosion rate increases, and the potential service life of the base pipe with the nozzle-type ICD decreases. The authors, having analyzed the changes in the pattern of flow velocity distribution after changing the angle of inclination of the channel of the nozzle-type ICD, made the assumption that the angle of inclination of the nozzle-type ICD to the pipe axis should be reduced not toward the main flow in the pipe but in the opposite direction.

## 5. Conclusions

1. An increase in flow velocity in the near-wall area and, as a consequence, an increase in wall shear stress (WSS) can lead to an increase in the corrosion wear rate of the pipe wall. From this, we can conclude that in order to decrease the failure probability of downhole equipment (base pipe with a nozzle-type flow control device (ICD) installed on it), it is necessary to decrease the WSS of the base pipe in order to decrease the base pipe corrosion rate.
2. Making some changes in the design and installing the inflow control devices on the base pipe can reduce the WSS level of the base pipe. Possible ways to reduce base-pipe WSS after a nozzle-type ICD are reducing the angle of the channel from 90 degrees to the axis of the base pipe in the opposite direction to the direction of the main flow and the mutual positioning of ICD nozzles in the base pipe (for example, opposite each other).
3. Although the studies determined the influence of WSS on the corrosion rate and made assumptions about the mechanisms of corrosion wear (the ratio of general and local corrosion at different WSSs; destruction of the sulfide film at high WSS), the influence of erosion factors on corrosion wear processes should be clarified.
4. At present, the authors have certain technical limitations on measuring the electrochemical potential at high temperatures and pressures. To conduct such studies, authors should carry out a modification of the rotating cage autoclave (RCA) installation and the adjustment of test methodology and ways of measuring WSS (direct measurement of WSS during the experiment on the RCA).

## Figures and Tables

**Table 1:** Effect of operation of downhole equipment on the parameters of corrosion and erosion processes.

	Downhole Equipment					
Factors Affecting Corrosion and Erosion Processes	Packer	Sand Screen	Inflow Control Devices	Centralizers	Shoe	Casing
The proportion of water in the stream	-	-	x	-	x/-	-
Presence and concentration of CO_2_ and H_2_S	-	-	-	-	-	-
Type of flow (bubble, projectile, etc.)	-	x	x	x	-	-
Wall shear stress (WSS)	-	-	x	-	-	x
The volume fraction of mechanical impurities in the stream	-	x	x	-	-	-
Particle sizes of mechanical impurities	-	x	-	-	-	-
Flow rates	-	-	x	x	-	-

“x”—downhole equipment affects the factor, “-“—downhole equipment does not affect the factor.

**Table 2:** Properties by reservoir.

Parameters	Dimension	Values
Bottom hole pressure	MPa	6.5–7.0
Reservoir temperature	°C	63–65
pH		7.8–7.9
Partial pressure of CO_2_	MPa	0.2

**Table 3:** Composition of the model water phase.

Component	Content, g/L
Na_2_SO_3_	0.08
NaHCO_3_	0.57
CaCl_2_·2H_2_0	0.30
MgCI_2_	0.07
NaCI	3.40
KCI	0.34
Mineralization	4.68

**Table 4:** The values of the maximum (local) WSS for various operation options.

Operating Variant	WSS, Pa	The Value of the Rotation Speed of the Stand in the Autoclave, rpm
Pipe without fitting	1	50
Pipe with fitting 90 degrees to the pipe axis	13	500
Pipe with fitting 45 degrees to the pipe axis	36	720

**Table 5:** Modified RCA method autoclave test results.

WSS, Pa	Rotational Speed	Sample	Mass Loss, g	Corrosion Rate (CR), mm/y	Av. Corrosion Rate (CR), mm/y
1	50 rpm	3	0.0639	0.391	0.443
		4	0.0769	0.470	
		8	0.0762	0.466	
13	500 rpm	2	0.1903	1.164	1.025
		4	0.1475	0.902	
		10	0.1646	1.007	
36	720 rpm	1	0.6638	4.061	4.000
		7	0.6979	4.270	
		8	0.6001	3.671	

**Table 6:** Summary table of accident-free liner life with installed ICDs.

Operating Variant	Rotational Speed	Av. Corrosion Rate, mm/y	The Period until Complete Corrosive Wear of the Pipe Wall with a Thickness of 7.4 mm
Pipe without fitting	50 rpm	0.443	16.7 y
Pipe with fitting 90 degrees to the pipe axis	500 rpm	1.025	7.2 y
Pipe with fitting 45 degrees to the pipe axis	720 rpm	4.000	1.85 y

**Figure A1:**
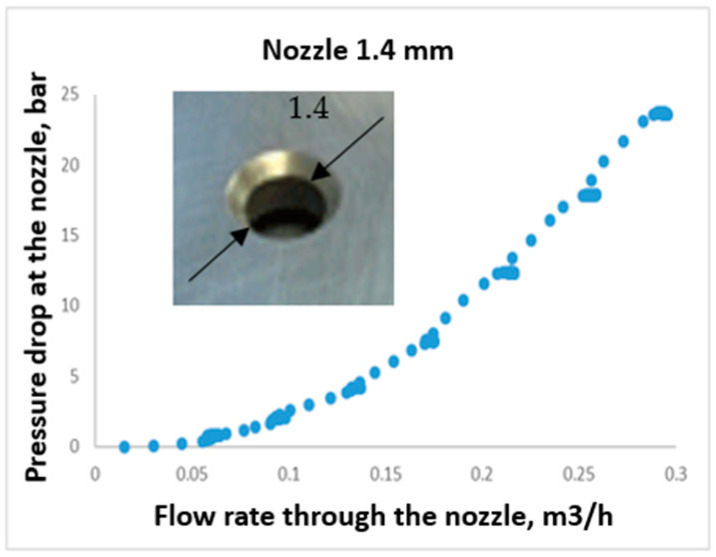
The example of the obtained hydrodynamic characteristics of the ICD.

**Figure A2:**
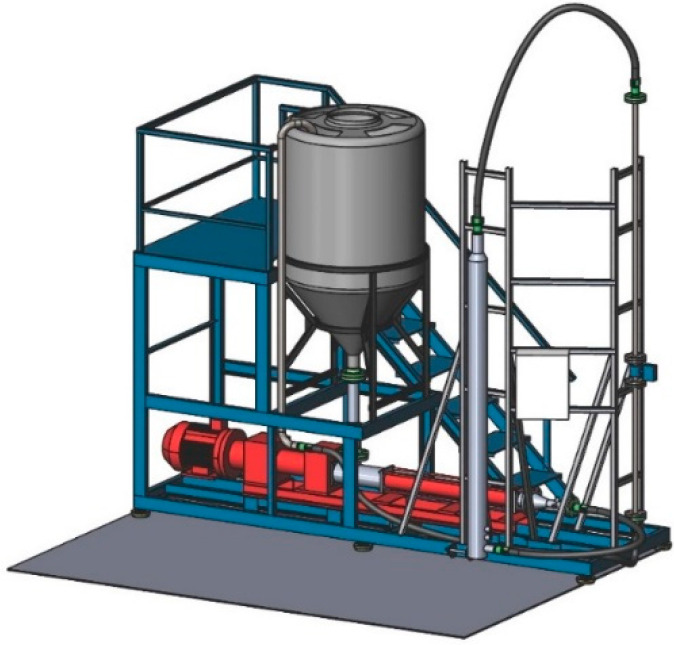
Hydraulic flow loop.

## Data Availability

The data presented in this paper are available on request from the corresponding author.

## Appendix A. Calculation of Operating Modes of Inflow Control Devices in the Well

To simulate the operation of the nozzle-type inflow control device (ICD) in the well, calculations were made of the operation of the nozzle-type ICD from a certain field in the territory of the Russian Federation (field “N”), where there is a high content of CO_2_ in the flow of produced oil. ICDs on the wells of this field can potentially be installed to combat the rapid water cut of the wells.

Calculations were made based on the Joshi formula (1,2) [9] considering the PVT properties of fluids and diagrams of the relative phase permeability of the “N” field.

The hydrodynamic characteristics of the nozzle-type ICD (Figure A1) for calculations were obtained during full-scale tests on a hydraulic flow loop (Figure A2).

The calculation was carried out by discretizing the well into sections with the nozzle-type ICD. The selection of pressure drops in each of the sections with the nozzle-type ICD to reduce local pressure drops to the total bottom hole pressure was carried out using the generalized gradient method in MS Excel.

Data on the calculation of the parameters of the nozzle-type ICD for the well “XXX” from field “N” are presented in Table A1.

**Table A1:** The calculation of the parameters of the nozzle-type ICD for the well “XXX” from field “N”.

Characteristic	Value	Dimension
Pipe inner diameter	99.5	mm
Pipe wall thickness	7.4	mm
Fluid viscosity	6.0	cP
Fluid density	998.0	kg/m^3^
Nozzle inlet speed	0.53233	m/s
Speed in the liner ahead of the nozzle	0.662	m/s
